# Supplementary material for: Intercalated water layers promote thermal dissipation at bio–nano interfaces
Source: Nat Commun. 2016 Sep 23;7:12854. doi: 10.1038/ncomms12854 (PMC5036148; doi:10.1038/ncomms12854)
Supplement: Supplementary Information — Supplementary Figures 1-5, Supplementary Notes 1-2 and Supplementary References [file ncomms12854-s1.pdf]

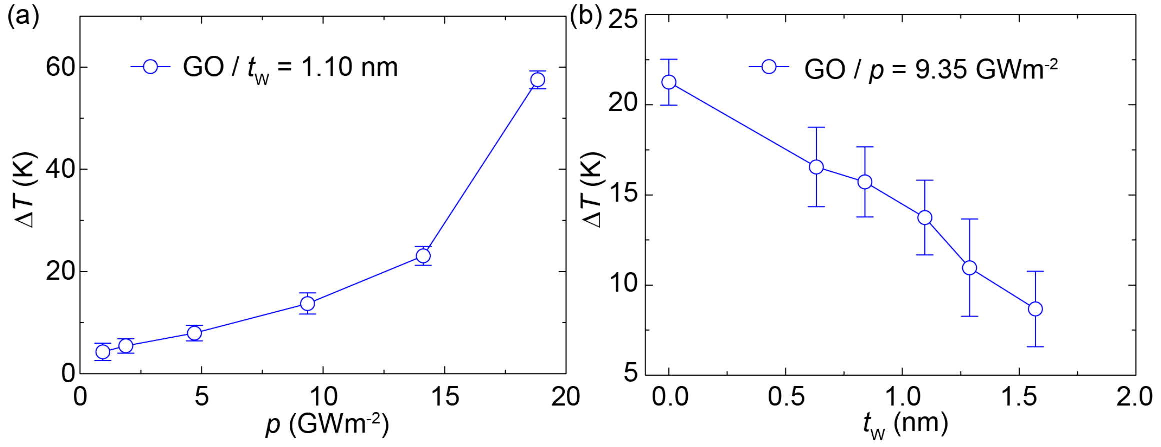

Supplementary Figure 1. Temperature rise in lipid bilayers upon heat generation in graphene oxide sheets. (a) Temperature rise in lipid bilayer after heat flux with power density p is injected into GO. (b) Temperature rise in the lipid bilayer as GO is heated at a power density of p = 9.35 GWm^{-2}, plotted as a function of the thickness of intercalated water layer.

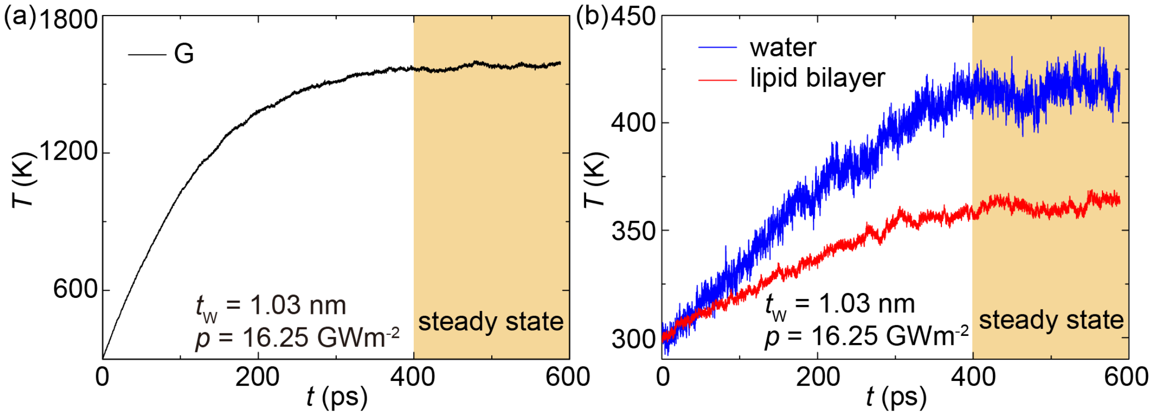

Supplementary Figure 2. Temperature evolution in the bio-nano hybrid. The steady-state temperature profile and temperature evolution obtained from the MD simulations. The simulations take about 400 ps to reach the steady state.

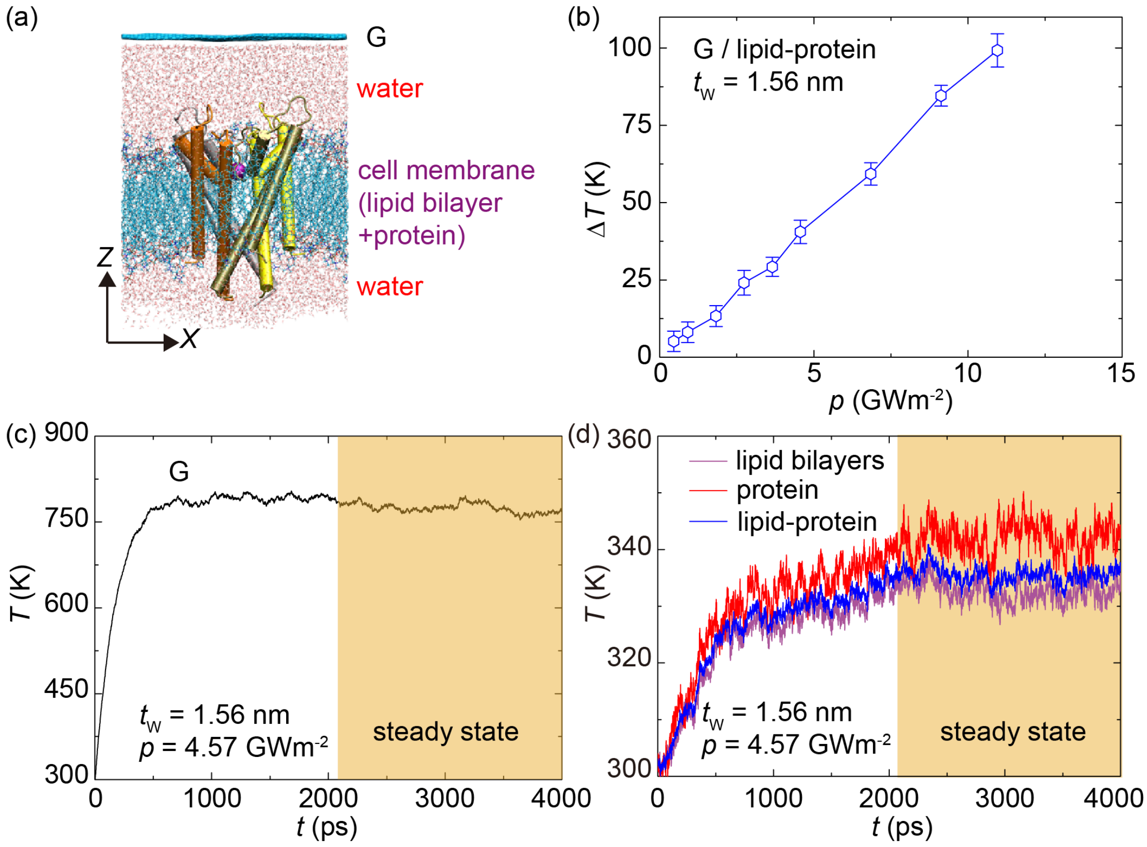

Supplementary Figure 3. Simulation results for the bio-nano interface with protein embedded in lipid bilayers. (a) Molecular model of a graphene/cell membrane interface with a potassium channel protein embedded in the lipid bilayer. The model also contains 13 Cl^- ions to maintain the charge-neutrality.[1] A 7.20 × 7.25 nm^2 supercell is constructed with periodic boundary conditions along the interface. The CHARMM36 forcefield is used for the protein, and other settings are the same as those used for the graphene/lipid bilayer interfaces. (b) Temperature rise in the lipid bilayer after a heat flux with power density p is injected into graphene. (c-d) The evolution of temperature in the MD simulations, which reaches a steady state after ~2 ns. This result shows that the protein has higher temperature than the lipid membrane. This inhomogeneity may explain the slightly lower critical power predicted for the graphene/lipid-protein interface (panel b) than the graphene/lipid interface but the difference is moderate (**Fig. 3a**).

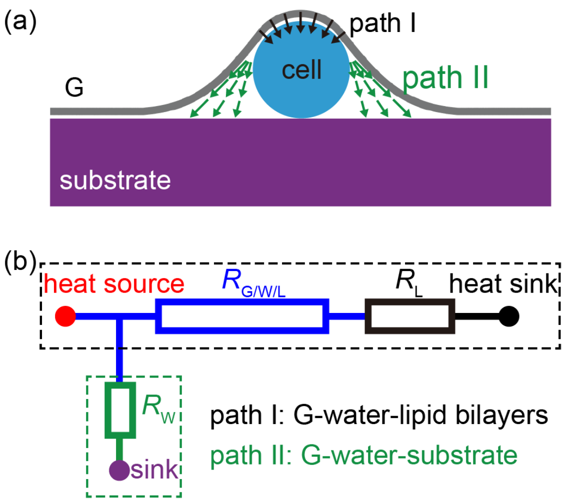

Supplementary Figure 4. Illustration of the model to predict heat dissipation across the bio-nano interface. Thermal dissipation pathways in a typical setup of bio-nano devices, where a cell is coated by graphene on a substrate. The thermal resistance of water is $R_W = L_W/\kappa_W A_1$, where L_W and A_1 are the effective length and interfacial area of water measured from intercalated water layer to the substrate (lipid bilayer). κ_W is the thermal conductivity of water. The thermal resistance of graphene/water/lipid (G/W/L) interface is $R_{G/W/L} = 1/G_C A_2$, where G_C is the ITC measured from our MD simulations, and A_2 is the area of G/W/L interface. For simplicity, here we assume $A_1 = A_2$ and $L_W = 0.75d$, where d is the diameter of cell. Considering a typical value of $d = 1$ μm, we obtain a ratio η between thermal resistance of path II and I as $\eta = R_W/(R_{G/W/L}+R_L) \approx R_W/(R_{G/W/L}) = G_C/(\kappa_W/L_W)$. With $\kappa_W = 0.563$ $Wm^{-1}K^{-1}$, $G_C = \sim 13.73\text{-}49.1$ $MWm^{-2}K^{-1}$ (from our MD simulations), we calculate the value of η in the range of 18.29-65.41, which suggests that the resistance of path II is one order larger than that of path I. Consequently, we limit our discussion on path I in this work, which is the major pathway for thermal dissipation.

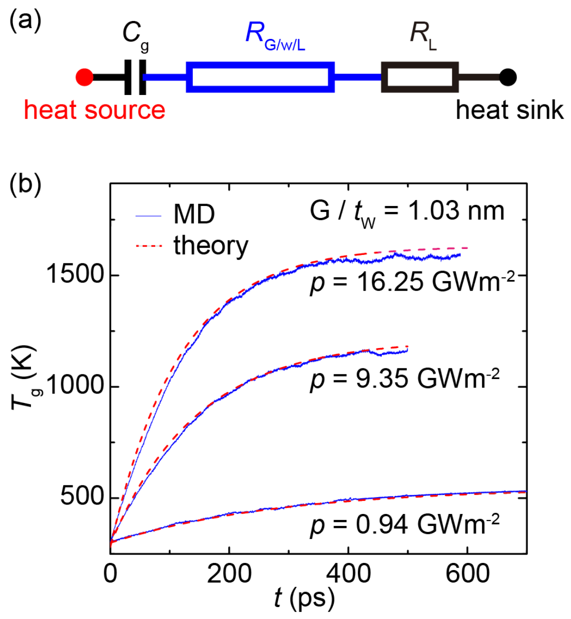

Supplementary Figure 5. Model predictions and simulation results for temperature evolution in the bio-nano hybrid. (a) Transient heat dissipation process of the hybrid graphene-water/lipid system, where C_g is the heat capacitor of graphene, $R_C = R_{G/W/L} + R_L$ is the thermal resistance of the graphene-water/lipid interface. Heat with power density p is injected into the system at heat source. Water on the other side of lipid bilayer is in contact with a thermal bath at 300 K, as the sink. (b) Temperature evolution in graphene with t_W = 1.03 nm under different power density p. The results obtained from MD simulations can be well predicted using the theoretical model (**Eq. 5**).

Supplementary Note 1

References for the Interfacial Energy Data in Fig. 4(b)

Carbon nanotube (CNT)/sodium-dodecyl-sulfate (SDS),[2] Pt/water,[3] graphene/oil,[4] graphene/phenolic resin,[5] graphene/polyethylene (PE),[4] CNT/Pt,[6] graphite/metal,[7, 8] diamond/Ti,[9] graphene/SiO_2,[10] graphene/SiC,[5] copper/SiO_2,[11] graphene/Cu(Ni),[12] benzene junction/diamond,[13] TiN/MgO,[14] Au/self-assembled monolayer (SAM)/quartz,[15] octane/water[16], surfactant/water[16] and protein/water.[17, 18]

Supplementary Note 2

A Model for the Transient Heat Dissipation Process

For both the transient and steady processes of heat dissipation through the lipid bilayer-graphene interface, we construct here a network model with thermal resistors and a heat capacitor, as illustrated in **Supplementary Fig. 5(a)**. We introduce Q_0 as the heat injection, Q_1 as the heat that leads to temperature change in graphene, and Q_2 as heat dissipation via the interfacial thermal resistance. From the definition and energy conservation, we have

$$Q_0 = \int pA\mathrm{d}t \tag{1a}$$

$$Q_1 = \int c_g\rho_g V_g \mathrm{d}T_g(t) \tag{1b}$$

$$Q_2 = \int G_C A(T_g(t)-T_e)\mathrm{d}t \tag{1c}$$

$$Q_0 = Q_1 + Q_2 \tag{2}$$

Here p is the power density, A is area of the bio-nano interface, c_g, ρ_g and V_g are the specific heat capacity, mass density and volume of graphene. $d = 0.34$ nm is the nominal thickness of graphene defined by its van der Waals diameter ($d = V_g/A$). $T_g(t)$ is the temperature in graphene at time t, $T_e = 300$ K is the temperature of water bath at the other side of lipid-bilayer, and $G_C \approx 1/R_C$ is the interfacial thermal conductance in the hybrid system. Thermal resistance of lipid bilayer is neglected here because it is far lower than the interfacial resistance.

By substituting **Eq. 1** into **Eq. 2**, we have

$$c_g\rho_g V_g \mathrm{d}T_g(t)/\mathrm{d}t + G_C A(T_g(t)-T_e) = pA \tag{3}$$

By defining the temperature difference between graphene and water bath at time t as $\theta_g(t) = T_g(t)-T_e$, we can simplify the equation and its initial condition as

$$\theta_g^{'}(t) + (G_C/c_g\rho_g d)\theta_g(t) = p/c_g\rho_g d \tag{4a}$$

$$\theta_g(t = 0) = 0 \tag{4b}$$

Eq. 4 can be solved by using the Laplace transform. The solution for temperature in graphene is

$$T_g(t) = T_e + p/G_C - p/G_C \exp(-tG_C/c_g\rho_g d) \quad (5)$$

To validate this theoretical model, we fit $T_g(t)$ through **Eq. 5** using our MD simulation results at different power densities. The results shown in **Supplementary Fig. 5(b)** confirm that the model predict well the simulation results, from the heating power p and interfacial thermal conductance G_C.

Supplementary References

1. Williamson MI, Alvis JS, East MJ, Lee GA. The potassium channel KcsA and its interaction with the lipid bilayer. *Cell. Mol. Life Sci.* **60**, 1581-1590 (2003).
2. Duan WH, Wang Q, Collins F. Dispersion of carbon nanotubes with SDS surfactants: A study from a binding energy perspective. *Chem. Sci.* **2**, 1407-1413 (2011).
3. Błoński P, López N. On the adsorption of formaldehyde and methanol on a water-covered Pt(111): A DFT-D study. *J. Phys. Chem. C* **116**, 15484-15492 (2012).
4. Katsumasa K, Susumu O. Energetics and electronic structures of alkanes and polyethylene adsorbed on graphene. *JPN J. Appl. Phys.* **52**, 06GD10 (2013).
5. Wells GH*, et al.* Determination of the adhesion energy of graphene on SiC(0001) via measurement of pleat defects. *Appl. Phys. Lett.* **105**, 193109 (2014).
6. Sen FG, Qi Y, Alpas AT. Improvement of the Pt/graphene interface adhesion by metallic adatoms for fuel cell applications. *Mater. Res. Soc. Proc.* **1213**, null-null (2009).
7. Schmidt AJ, Collins KC, Minnich AJ, Chen G. Thermal conductance and phonon transmissivity of metal-graphite interfaces. *J. Appl. Phys.* **107**, 104907 (2010).
8. Vanin M, Mortensen JJ, Kelkkanen AK, Garcia-Lastra JM, Thygesen KS, Jacobsen KW. Graphene on metals: A van der Waals density functional study. *Phys. Rev. B* **81**, 081408 (2010).
9. Guo H, Qi Y, Li X. Adhesion at diamond/metal interfaces: A density functional theory study. *J. Appl. Phys.* **107**, 033722 (2010).
10. Wei G, Penghao X, Graeme H, Kenneth ML, Rui H. Interfacial adhesion between graphene and silicon dioxide by density functional theory with van der Waals corrections. *J. Phys. D Appl. Phys.* **47**, 255301 (2014).
11. Nagao K, Neaton JB, Ashcroft NW. First-principles study of adhesion at Cu/SiO_2 interfaces. *Phys. Rev. B* **68**, 125403 (2003).
12. Xu Z, Buehler M, J. Interface structure and mechanics between graphene and metal substrates: A first-principles study. *J. Phys. Condens. Matter* **22**, 485301 (2010).

13. Wang Y, Xu Z. The critical power to maintain thermally stable molecular junctions. *Nat. Commun.* **5**, (2014).
14. Costescu RM, Wall MA, Cahill DG. Thermal conductance of epitaxial interfaces. *Phys. Rev. B* **67**, 054302 (2003).
15. Losego MD, Grady ME, Sottos NR, Cahill DG, Braun PV. Effects of chemical bonding on heat transport across interfaces. *Nat. Mater.* **11**, 502-506 (2012).
16. Rivera JL, McCabe C, Cummings PT. Molecular simulations of liquid-liquid interfacial properties: Water-n-alkane and water-methanol-n-alkane systems. *Phys. Rev. E* **67**, 011603 (2003).
17. Morozenko A, Leontyev IV, Stuchebrukhov AA. Dipole moment and binding energy of water in proteins from crystallographic analysis. *J. Chem. Theory Comput.* **10**, 4618-4623 (2014).
18. Romanczyk PP, Radon M, Noga K, Kurek SS. Autocatalytic cathodic dehalogenation triggered by dissociative electron transfer through a C-H…O hydrogen bond. *Phys. Chem. Chem. Phys.* **15**, 17522-17536 (2013).
